# Neutrophils mediated multistage nanoparticle delivery for prompting tumor photothermal therapy

**DOI:** 10.1186/s12951-020-00682-7

**Published:** 2020-09-29

**Authors:** Bo Ye, Bao Zhao, Kun Wang, Yilong Guo, Qinguo Lu, Longpo Zheng, Ang Li, Jianou Qiao

**Affiliations:** 1 Department of Thoracic Surgery, Shanghai Chest Hospital, Shanghai Jiao Tong University, Shanghai, China; 2 Department of Otorhinolaryngology Head and Neck Surgery, First Affiliated Hospital of Bengbu Medical College, Bengbu, China; 3 Cancer Center, Shanghai East Hospital, Tongji University, Shanghai, China; 4 Department of Thoracic Surgery, Pizhou people’s hospital, Xuzhou Medical University, Pizhou, China; 5 Shanghai Tenth People’s Hospital, School of Medicine, Tongji University, Shanghai, 200072 People’s Republic of China; 6 School of Life Science and Technology, Tongji University, 1239 Siping Road, Shanghai, 200092 People’s Republic of China; 7 Department of Respiratory Medicine, Shanghai Ninth Peoples Hospital, Shanghai Jiao Tong University School of Medicine, 639 Zhizaoju Rd, Shanghai, 200011 China

**Keywords:** Neutrophils, Photothermal therapy, Inflammatory signal, Au nanorod, Tumour homing

## Abstract

**Background:**

Neutrophil-based drug delivery system possesses excellent advantages in targeting at tumour because neutrophils are easily recruited by chemotactic factor in tumor microenvironment. Herein, we developed a novel tactic of multistage neutrophils-based nanoparticle delivery system for promoting photothermal therapy (PTT) of lung cancer.

**Results:**

Au nanorod (AuNR) was successfully modified with bovine serum albumin (AuNRB) and further conjugated with RGD (AuNRBR), followed by neutrophil internalisation to obtain neutrophils-based delivery system (AuNRBR/N). The engineered neutrophils efficiently migrated across the epithelial cells due to inflammatory signal. They exhibited better toxicity against Lewis cells with laser irradiation in vitro. Moreover, AuNRBR/N showed significantly more targetability to tumour tissue compared with cell carrier-free AuNRBR, as demonstrated in Lewis tumour-bearing mice. The enhanced tumour homing efficiency of AuNRBR/N together with subsequently released AuNRBR from the neutrophils was favourable for further deep tissue diffusion and contributed to the inhibition of the tumour growth in PTT and improved survival rate (over 120 days).

**Conclusions:**

Overall results illustrated that the design of cell-based nanoparticle delivery system for PTT of cancer is promising. 
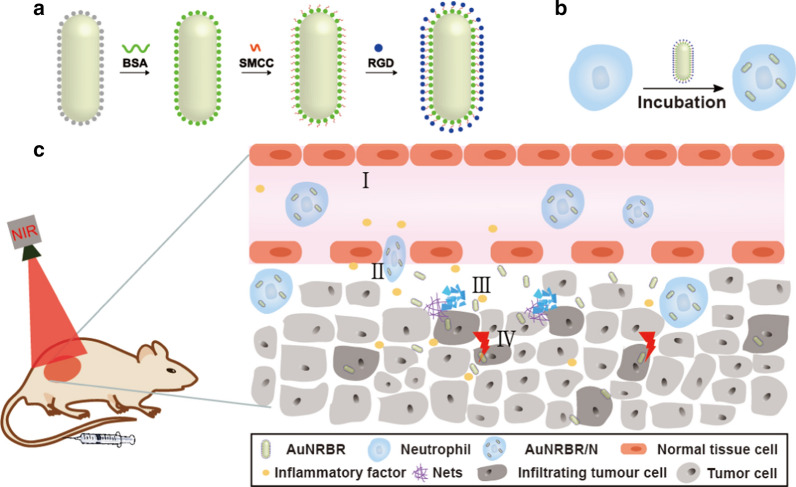

## Background

Photothermal therapy (PTT) is a promising treatment modality for the improvement of cancer therapy, especially for some epidermomas, such as melanoma and breast cancer. Photosensitisers are typically used to generate hyperthermia for the elimination of tumour under near-infrared (NIR) irradiation whilst minimising thermal diffusion to normal tissue. Au nanorod (AuNR) with strong NIR absorption was widely studied recently for the presence of plasmonic nanostructures, which serve as highly localised heat sources under NIR irradiation through the photothermal effect [1]. However, systemic administration of AuNR faced many challenges, including poor distribution in vivo and less efficient accumulation within the tumour, which lead to undesired hyperthermia.

Nanoparticles suffer from rapid clearance by the mononuclear phagocytic system (MPS) mostly resident in the liver, thereby effectively impeding the circulation of these nanoparticles in blood and accumulation in tumour [2]. Warren Chan and colleagues reported that only 0.7% of nanoparticles were successfully delivered to tumour site [3], and this was the main hurdle for the application of AuNR in PTT. The strategy for improving nanoparticle accumulation at tumour has been vigorously pursued. For example, the widely accepted approach to increase in vivo circulation time of AuNR was to modify polyethylene glycols (PEGs) [4]. However, the detection of anti-PEG IgG and IgM indicated accelerated blood clearance of PEG-based AuNR delivery systems from body and hypersensitivity reactions entailing fatal anaphylaxis. Besides, AuNR was modified with targeting molecule (such as folate, RGD, peptide, aptamers and tumour specific antibody) [5–8] to realize the active targeting on tumour. Nevertheless, antibodies and aptamers usually suffer from poor specificity, unfixed configuration and digestion by protease and nucleases. And thus, these strategies did not overcome the limitation of low accumulation in the tumour through its leaky, hastily built vasculature and subsequent poor penetration deeply into the tumour tissue [9].

Cell-based nanoparticle delivery system has achieved great progress in recent years and is another feasible alternative for augmenting tumour targeting. Immune cells, such as monocyte [10], macrophage [11], T cell [12] and neutrophil [13, 14] were utilised to load or conjugate with therapeutic agents for antitumour therapy. Among these cells, neutrophils can be recruited by chemoattractive agents produced by tumour tissue; which result in the ability of neutrophils to penetrate into the tumour tissue [13, 15]. When loaded with therapeutic agents, neutrophils act as excellent Trojan horses and could pass across the endothelial barrier and release the cargoes in tumour tissues specifically stimulated by inflammation factors [16]. Neutrophils are used to load liposome-carrying PTX or doxorubicin-loaded magnetic mesoporous silica nanoparticles for the therapy of postoperative malignant glioma [13, 17]. In consequence, neutrophil-based delivery system was a good alternative for improving nanoparticle accumulation and penetration in tumour.

**Fig. 1:**
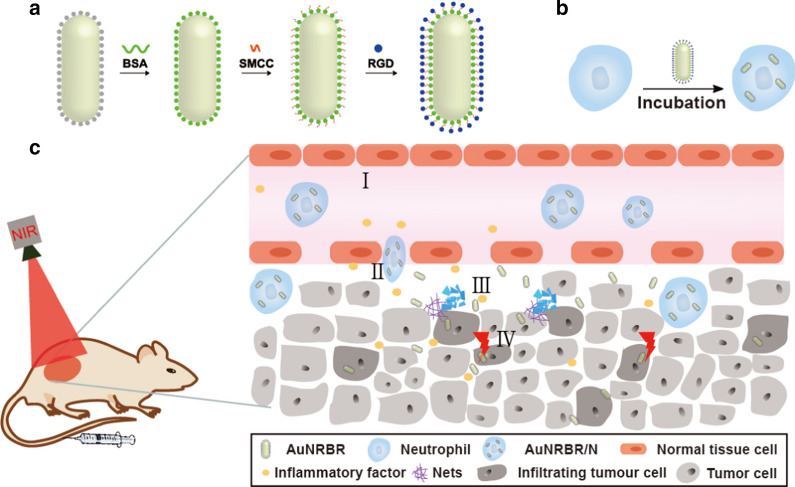
Schematic illustration of **a** synthesis of AuNRBR, **b** the preparation of neutrophil ingesting AuNRBR and **c** in vivo PTT: (I) AuNRBR/N recruited by tumor; (II) AuNRBR/N passing across the tissue; (III) release of AuNRBR from AuNRBR/N along with NET formation; (IV) AuNRBR penetrating into the tumor tissue deeply to produce an antitumor effect under laser irradiation

Inspired by this, we constructed a novel cell-based AuNR delivery system by loading AuNR into neutrophils (Fig. 1a, b). The synthetic AuNR was modified with BSA (AuNRB) to promote the biocompatibility that was subsequently internalised by neutrophil. Furthermore, AuNRB was further conjugated with RGD to increase penetration in tumour site after neutrophil delivered AuNRBR, which exhibited good photothermal property, internalisation and toxicity to mouse lung adenocarcinoma Lewis cells in vitro efficiently. In addition, neutrophils carrying AuNRBR could obviously migrate across the epithelial cells due to recruitment by chemotactic factor leading to the promoted targeting efficiency of tumour in vivo. They also exhibit potent inhibition effect on Lewis tumour to enhance the survival rate of mice (Fig. 1c).

## Methods

### Materials

Hydrochloric acid (HCl, 37%) was purchased from Sinopharm Chemistry. Foetal bovine serum (FBS), RPMI Media 1640, Dulbecco’s modified Eagle’s medium (DMEM), penicillin–streptomycin and trypsin were supplied by Gibco Invitrogen. HS-RGD was purchased from GL Biochem (Shanghai), Ltd. FITC-conjugated anti-mouse CD11b and PE/Cy7-conjugated anti-mouse Ly-6G/Ly-6C were purchase from Tonbo Biosciences. CCK8 and 4,6-diamidino-2-phenylindole (DAPI) were obtained from Beyotime Institute of Biotechnology. Paraformaldehyde (4%) was obtained from DingGuo Chang Sheng Biotech. Gold (III) chloride trihydrate (HAuCl_4_^·^3H_2_0), 4-(N-Maleimidomethyl) cyclohexane-1-carboxylic acid 3-sulfo-N-hydroxysuccinimide ester sodium salt (sulfo-smcc) and bovine serum albumin (BSA) was purchased from Sigma-Aldrich (Shanghai) Trading. Phosphate and phosphate buffered saline (PBS, pH = 7.2, 10 mM) was obtained from Hyclone. Hexadecyl trimethyl ammonium bromide (CTAB), silver nitrate concentrate (AgNO_3_), ascorbic acid, sodium borohydride (NaBH_4_), dimethyl sulfoxide (DMSO), Fluorescein5(6)-isothiocyanate (FITC) and formylmethionylleucylphenylalanine (fMLP) were supplied by Aladdin. The BODIPY NHS Ester (succinimidyl ester) and DiD were supplied by Thermo Fisher. Lewis cell line was purchased from American Type Culture Collection (Manassas, VA) and maintained according to American Type Culture Collection-recommended conditions.

### Characterisation

Fluorescence spectra were recorded by a Hitachi F2500 luminescence spectrometer (Hitachi, HongKong). UV–Vis spectra were characterised by an UV–vis spectrophotometer (Varian, Hong Kong). Transmission electron microscope (TEM, Tecnai-12 Bio-Twin, FEI, Netherlands) was used to observe the nanoparticle morphology. The zeta potential was determined by Zeta Sizer Nano ZS (ZS90, Malvern). Cell viability assay was conducted by using a microplate reader. The cellular uptake was detected using confocal laser scanning microscopy (CLSM, Nikon A1R, Japan). Flow cytometer (FCM, BD FACSVerse) was performed to quantitatively analyse the cellular uptake of nanoparticles. The infrared thermal camera (DALI TECHNOLOGY, LT3-P) was used to record the temperature of the dispersions. The inductively coupled plasma-atomic emission spectroscopy (ICP-AES, iCAP 7400) was used to determine Au concentration.

### Synthesis of AuNR, AuNRB and AuNRBR

AuNR was synthesised by the seeded growth according to the reported the literature with little modification [20]. AuNRB was got by modifying AuNR with BSA based on the method described by Moustafa et al. [21]. Briefly, 1 mL BSA solution dissolved in the PBS (pH = 7.2–7.4) at a concentration of 0.25 mM was added to 10 mL of the AuNR solution (0.3 nM), and the mixed solution was stirred vigorously for 12 h at room temperature. Then, the reaction was stopped *by* removing the excess BSA by centrifugation at 12,000 rpm for 5 min and the precipitate was resuspended in ultrapure water to gather AuNRB.

AuNRB was suspended in PBS (2 mg/mL) were respectively functionalised by sulfo-smcc (150 μL, 5 mg/mL) to endow AuNRB with Mal for further conjugation with HS-RGD. The functionalisation was performed at room temperature (RT) for 1 h. Then, the excessive Sulfo-SMCC was removed by centrifugation with speed of 10,000 rpm for 10 min thrice. The purified solution was collected and 100 μL HS-RGD (25 mg/mL) was added by stirring at 350 rpm at RT for 2 h. Afterwards, the reaction was stopped by removing the excess RGD by centrifugation with speed of 10,000 rpm for 10 min for 3 times and the solution was resuspended in ultrapure water with final volume of 2 mL. The sample was stored at 4 °C for further study.

FITC DMSO solution was reacted with BSA at a molar ratio of 3: 1 (FITC: BSA) for 6 h. After the ultrafiltration with water to remove free FITC, FITC labelled BSA was collected and used to synthsize FITC-AuNRBR and FITC-AuNRB.

### In vitro photothermal effect

AuNRBR dispersed in PBS with the concentration of 10, 20, 40 and 80 μg/mL in a tube was irradiated with an 808 nm laser at the power density of 1 W/cm^2^ for 8 min. The temperature increase was recorded at 1, 2, 3, 4, 5, 6, 7 and 8 min, and the thermal images of the dispersions were recorded at 8 min by an infrared thermal camera, respectively. PBS was used as control treated under the same conditions.

### Isolation and purity of neutrophils from mice

Neutrophils were isolated from femurs and tibias of 3-week-old to 4-week-old C57BL/6 mice. Cells isolated from femurs and tibias were suspended in PBS and centrifuged at 1000 rpm for 3 min. The collected cells were resuspended with 2 mL separation mixed solution containing 55%, 65% and 78% (v:v) Percoll in PBS, followed by centrifugation for 30 min at 500 g. Afterwards, neutrophils were collected at the interface of the 65% and 78% fractions and further washed by ice-cold PBS thrice.

To identify the purity of neutrophils, they were doubly stained with FITC-conjugated anti-mouse CD11b and PE/Cy7-conjugated anti-mouse Ly-6G/Ly-6C to analyse by flow cytometry. Neutrophils were also directly observed by microscope.

### AuNRBR loaded into neutrophils (AuNRBR/N)

AuNRBR was incubated with neutrophils to produce AuNRBR/N. To measure the loading efficiency, AuNRBR was labelled with FITC (1 μg/10^5^ cells) and incubated with neutrophils for 10, 30 and 60 min at 37 °C with slight shaking. Afterwards, the cells were centrifuged at 1200 rpm for 5 min to remove excess AuNRBR-FITC. Finally, the cells were analysed by flow cytometry and observed by fluorescence microscope.

### AuNRBR release from AuNRBR/N

The release behaviour of AuNRBR from AuNRBR/N was investigated by determining the amount of AuNRBR in supernatant. AuNRBR/N was resuspended in RPMI 1640, followed by irradiation with 808 nm laser. Then, the cell suspension was cultured with 5% CO_2_ at 37 °C. At the interval time point (0, 1, 2, 4, 8 and 12 h), the cell suspension was centrifuged at 2000 g for 5 min. The supernatant was collected, and the AuNRBR content was determined by ICP-AES. Additionally, AuNRBR/N suspension with no laser irradiation was treated with the same process used in the control.

### Cellular uptake of AuNRB and AuNRBR

Lewis cells were planted in 6-well plate with DMEM containing 10% FBS and incubated at 37 °C and 5% CO_2_. They were allowed to grow until about 70% confluency. Then, the medium was removed and replaced with 2.5 mL serum-free DMEM with FITC labelled AuNRB and AuNRBR (equivalent FITC, quantified by fluorescence spectra) and incubated for 4 h, respectively. After incubation, Lewis cells were washed twice with PBS and collected for uptake analysis by flow cytometry. To more directly observe the uptake of FITC labelled AuNRB and AuNRBR by Lewis cells, the same operation as above was performed except for the planting of Lewis cells in laser confocal dishes. The cells washed with PBS were fixed with 4% paraformaldehyde and counterstained with DAPI for further observation by confocal laser scanning microscopy (CLSM).

### Endothelial permeability

The permeability was determined by using a transwell system (5 μm pore size, EMD Millipore Corporation). Briefly, human umbilical vein endothelial cells (HUVECs) were seeded in the upper chamber of the transwell in the 24-well plate and cultured with complete medium. The tightness of the cell monolayer was determined by measuring the trans-endothelial electrical resistance (TEER) value. When the TEER value arrive at or beyond 200 Ω cm^2^, the cell monolayer was used for the transmigration studies.

AuNRBR/N was added into upper chamber to co-incubate with HUVECs for 4 h, as well as serum-free medium added into the lower chamber in the presence or absence of fMLP. Then, the media in the upper and lower chambers were collected, and the cell monolayer on the transwell membrane was harvested. The AuNRBR in the supernatant, intracellular and filtrate compartments was measured by ICP-AES to further calculate the ratio by comparing with the input amount of AuNRBR.

### Cell viability

Lewis cells were planted in 96-well plate with DMEM containing 10% FBS and incubated for 24 h. Afterwards, the medium was removed and replaced with 100 μL serum-free DMEM containing AuNRBR/N (Au concentration of 10, 20, 40, 60, 80 μg/mL) and incubated for 6 and 12 h, respectively. Then, the medium was removed and washed with PBS twice. Another 200 μL of DMEM was added prior to irradiation with or without an 808 nm laser for 5 min (0.5 W/cm^2^). Following incubation overnight at 37 °C, the cell viability was determined by the above mentioned CCK-8 protocol.

### In vivo tumour targeting

The mice (C57BL/6, 5–6 weeks, female) were injected subcutaneously with Lewis cells to build tumour-bearing mice model and divide into 3 groups (3 mice per group). When the tumour volume reached about 200 mm^3^, the fluorescence dye (BODIPY)-labelled AuNRBR and AuNRBR/N (equivalent fluorescence dye) were injected intravenously to the mice. At 4, 8, 12, 24 and 48 h post-injection, the images were taken. For further analysis of targeting efficiency, the major organs and tumours were taken out to image at 12 h post-injection. Additionally, the tumour tissues were fixed with paraformaldehyde (4%) and cut into 20 μm sections, untreated tumour-bearing mice tissue used as negative control. The slides were observed by CLSM only after staining with DAPI.

To further analyse the ratio of the administered neutrophils to total neutrophils in tumour, the AuNRBR/N stained with DiD was injected intravenously into tumour-bearing mice. Then, the mice were sacrificed at each individual time point (4, 8, 12, 24 and 48 h), and the tumour tissues were collected. Subsequently, the tumour tissues were digested to make a cell suspension, followed by staining with PE/Cy7-conjugated anti-mouse Ly-6G/Ly-6C and analysis by flow cytometry.

### In vivo photothermal effect

AuNRBR/N and AuNRBR dispersed in PBS at a concentration of 100 μg/mL were injected intravenously into tumour-bearing mice. After 12 h, the mice were irradiated with an 808 nm laser at the power density of 0.5 W/cm^2^ for 5 min. The temperature increase was recorded at 0, 1, 3 and 5 min, and the thermal images of the AuNRBR/N-treated mice were recorded. PBS was used as control and treated under the same conditions.

### In vivo antitumour therapy

The tumour-bearing mice models were built as described above. Twenty tumour-bearing mice were randomly divided into four groups with five mice per group and intravenously injected with AuNRBR/N, AuNRBR and AuNR with equivalent doses of Au (100 μg) every 3 days for 3 times per day. The mice injected with PBS were used as the control. The tumours of mice were exposed to 808 nm laser irradiation (0.5 W/cm^2^) for 5 min at 12 h post-injection. The tumour size was measured, and body weight was recorded every other day along with the day the mice died. The tumour volume was calculated through the formula: V = (L × W^2^)/2 (L, tumour length; W, tumour width). On day 15, the mice were sacrificed, and tumours were extracted for imaging and further fixed in 4% paraformaldehyde for H&E assay according to the protocol.

### Statistical analysis

The statistical significance of the differences between sphere groups was calculated using a one-way ANOVA test with SPSS 19.0 (SPSS Inc., USA). Significant differences between or among the groups are implied by *p < 0.05, **p < 0.01, ***p < 0.001.

## Results and discussion

### Synthesis and characterisations of AuNR, AuNRB and AuNRBR

**Fig. 2:**
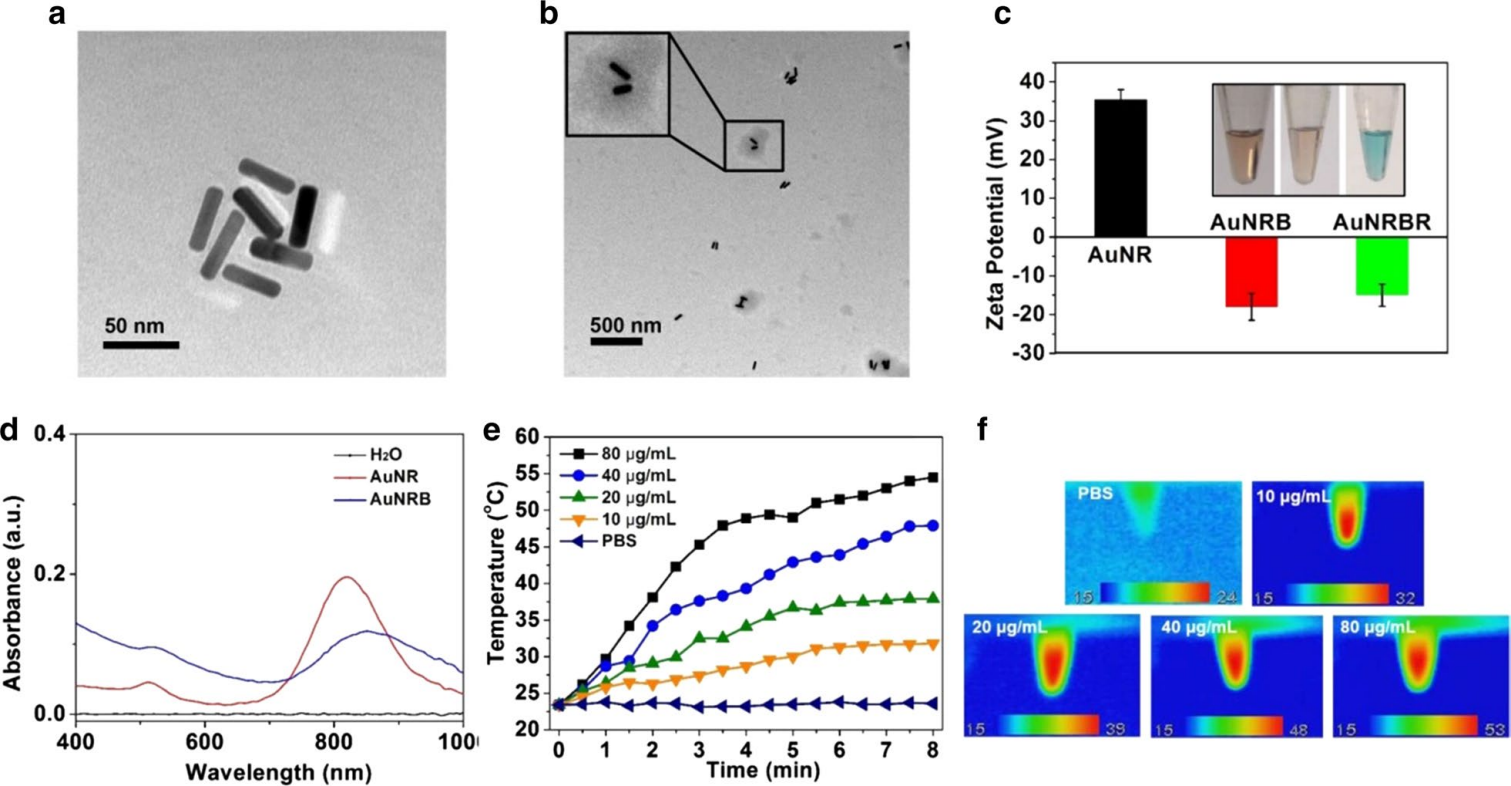
TEM of **a** AuNR and **b** AuNRB; **c** zeta potential of AuNR, AuNRB and AuNRBR, insertion of the images of the solution of AuNR, AuNRB and BODIPY labeled RGD conjugated with AuNRB. **d** UV measurement of AuNR and AuNRB. **e** Photothermal property of AuNRBR with different concentrations gradient. **f** In vitro infrared thermal images of various concentrations (0, 10, 20, 40 and 80 μg/mL) immediately after illumination. The error bars were presented as mean ± SD (n = 3)

AuNR was successfully synthesised with an average size of around 50 nm, as observed by TEM (Fig. 2a). The zeta potential of AuNR (Fig. 2c) was around + 35 mV, which was due to the cationic surfactant of CTAB. The AuNR was modified with BSA to increase biocompatibility, as demonstrated by the TEM (Fig. 2b). As shown in Fig. 2c, the zeta potential decreased to around − 18 mV compared with AuNR, thereby suggesting the successful conjugation of BSA to the surface of AuNR. To further identify the successful modification of BSA, UV was used to determine that the transverse and longitudinal peaks of AuNRB experienced redshift compared with AuNR (Fig. 2d), which was due to the plasmonic coupling of AuNR in this system. Moreover, AuNRB was conjugated with RGD by characterisation with the zeta potential of around − 17 mV (Fig. 2c). Besides, RGD labelled with BODIPY was conjugated to AuNRB along with the change of the solution colour from pink to blue after purification (Fig. 2c, insertion), thereby indicating that RGD was successfully conjugated to AuNRB. The photothermal property of AuNRBR was studied by using an 808 nm laser. Figure 2e shows that the temperature of the solutions rose was dependent on the concentration of AuNRBR. Interestingly, AuNRBR solutions (C_Au_ = 80 μg/mL) exhibited rather high temperature, which increased up to 50 °C within 5 min. In addition, the thermal images of AuNRBR had good photothermal conversion efficiency, thereby indicating the good photothermal property of AuNRBR due to the plasmonic coupling effect (Fig. 2f).

### Neutrophils loading AuNRBR and the release efficiency when irradiated with an 808 nm laser

**Fig. 3:**
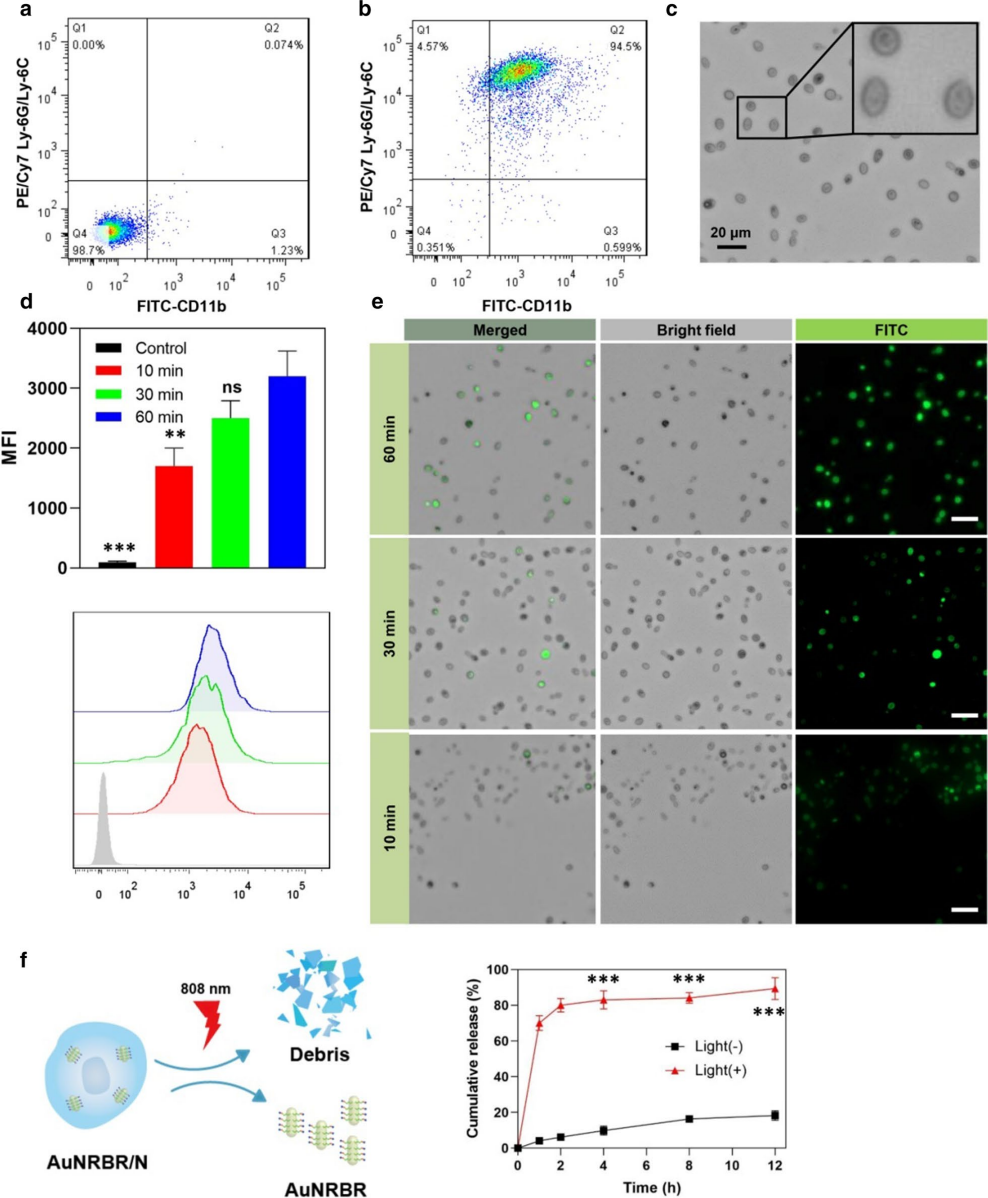
The purity determination of neutrophils **a** not stained or **b** doubly stained with FITC-CD11b and PE/Cy7 Ly-6G/Ly-6C analyzed by flow cytometry, respectively. The average population of the cells shown in the corner of each quadrant. **c** Images of purified neutrophil observed by microscope. **d**, **e** Neutrophils incubated with FITC-labeled AuNRBR for different time were determinated by flow cytometry and analyzed by mean fluorescence intensity **d** and observed by fluorescence microscope (**e**). **f** The release of AuNRBR from AuNRBR/N when irradiated with an 808 nm laser or not (n = 3). The error bars were presented as mean ± SD (n = 3). Scale bar: 20 μm. **p < 0.01 vs the 60 min group, ***p < 0.001 vs the Light (-) group

Firstly, the neutrophils were isolated from the mice bone marrow. The purified neutrophils were doubly stained with FITC-CD11b and PE/Cy7 Ly-6G/Ly-6C and analysed by flow cytometry. The yield was about 2.5 × 10^6^ cells/mouse, and the purity of neutrophils was approximately 95% (Fig. 3a, b). The morphology of the obtained neutrophils was observed directly under the microscope. They were polymorphonuclear and small, with sizes of approximately 8 μm (Fig. 3c).

Considering the short lifespan (only a few hours after isolation from blood) and quick phagocytosis ability of neutrophils, the incubation time was optimized to ensure the effective loading amount of AuNRBR. Figure 3d shows that neutrophils incubated with FITC-labelled AuNRBR showed more fluorescence signals with time going by according to flow cytometry analysis results. At the post-incubation time of 60 min, the fluorescence intensity was the highest, thereby suggesting that the ideal incubation time in which viability was not affected was 60 min. The neutrophils loading AuNRBR were also observed by CLSM. The same conclusion was drawn, i.e. neutrophils incubated with AuNRBR for 60 min showed stronger green fluorescence compared with those incubated for 10 min or 30 min, as observed by CLSM (Fig. 3e). Therefore, the incubation time of 60 min was particularly well suited to load AuNRBR into neutrophils.

To investigate AuNRBR release mediated by 808 nm laser irradiation, the content of AuNRBR was determined at each interval time. As shown in Fig. 3f, most AuNRBR remained in neutrophils, and only around 15% AuNRBR was detected in the medium at 12 h, thereby indicating that AuNRBR/N was stable in a normal physiological environment. However, a rather rapid release of AuNRBR from AuNRBR/N occurred when irradiated with an 808 nm laser. Approximately 80% AuNRBR was released from AuNRBR/N within 2 h. AuNRBR was capable of being released efficiently from AuNRBR/N under laser irradiation, thereby further exerting antitumour function.

### Cellular uptake and cytotoxicity of AuNRBR and AuNRBR/N

**Fig. 4:**
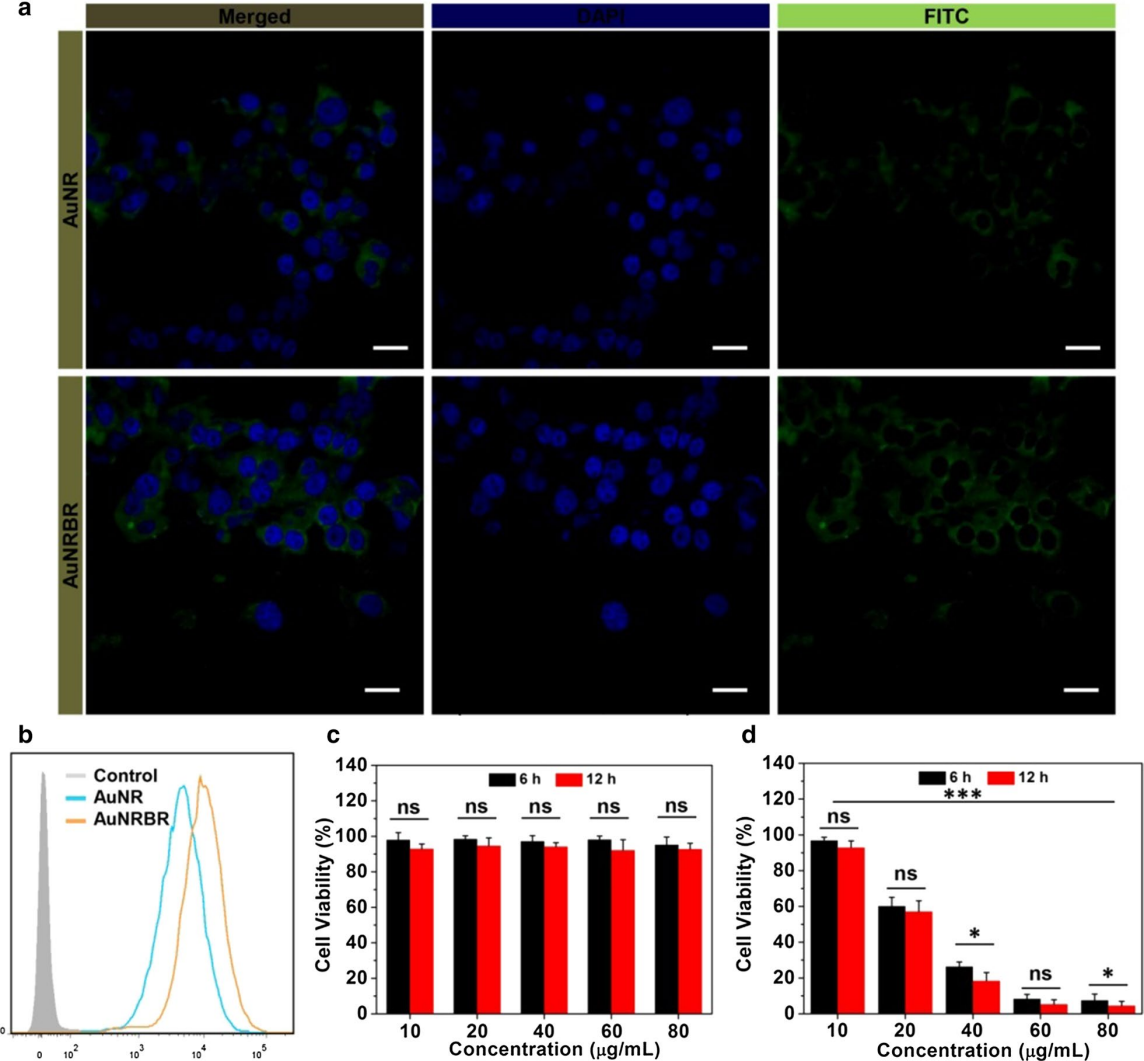
Lewis cells uptake determination via incubating FITC-labeled AuNR and AuNRBR for 4 h, respectively, **a** observed by CLSM. **b** quantified by flow cytometry. **c** Dark toxicity and **d** phototoxicity to Lewis cells incubated with AuNRBR/N including different concentrations of Au for 6 h and 12 h under laser, respectively. The error bars were presented as mean ± SD (n = 6). Scale bar: 20 μm. *p < 0.05 and ***p < 0.001

The cell uptake was estimated by using CLSM and flow cytometry to prove the targeting effect of AuNRBR. As shown in Fig. 4a, Lewis cells incubated with AuNRBR showed stronger fluorescence signals compared with AuNRB, as observed by CLSM. The cellular uptake was further measured quantitatively by flow cytometry. As shown in Fig. 4b, the mean fluorescence intensity of AuNRBR was much higher than that of AuNRB, which implied that the modification of RGD could increase the cellular uptake efficiently. The phenomenon above was due to the specific binding of RGD to integrin receptors expressed on Lewis cells [18].

Next, we detected the cytotoxicity of AuNRBR and AuNRBR/N to tumor cells with or without laser irradiation. As illustrated in Fig. 4c, AuNRBR/N exhibited little cytotoxicity to Lewis cells at 6 and 12 h post-incubation without laser irradiation, which demonstrated the excellent biosafety of AuNRBR/N without laser irradiation and selectivity for tumour ablation in PTT. In contrast, AuNRBR/N was able to inhibit the proliferation of Lewis cells effectively both at 6 and 12 h post-incubation with laser irradiation (Fig. 4d). Higher concentration of Au (40, 60 and 80 μg/mL) displayed stronger toxicity, in which Lewis cell viability was below 50% and close to around 10%. The potent cytotoxicity of AuNRBR/N was ascribed to the stronger photothermal effect of AuNRBR and extracellular trap net released from neutrophils [19]. Moreover, the cell viability of group AuNRBR/N at 12 h post-incubation decreased compared with that at 6 h post-incubation, indicating that more AuNRBR was delivered to Lewis cells with extended incubation time. Therefore, AuNRBR/N was capable of killing tumour cell in vitro with laser irradiation.

### AuNRBR/N promote endothelial permeability in vitro and tumour targeting in vivo

**Fig. 5:**
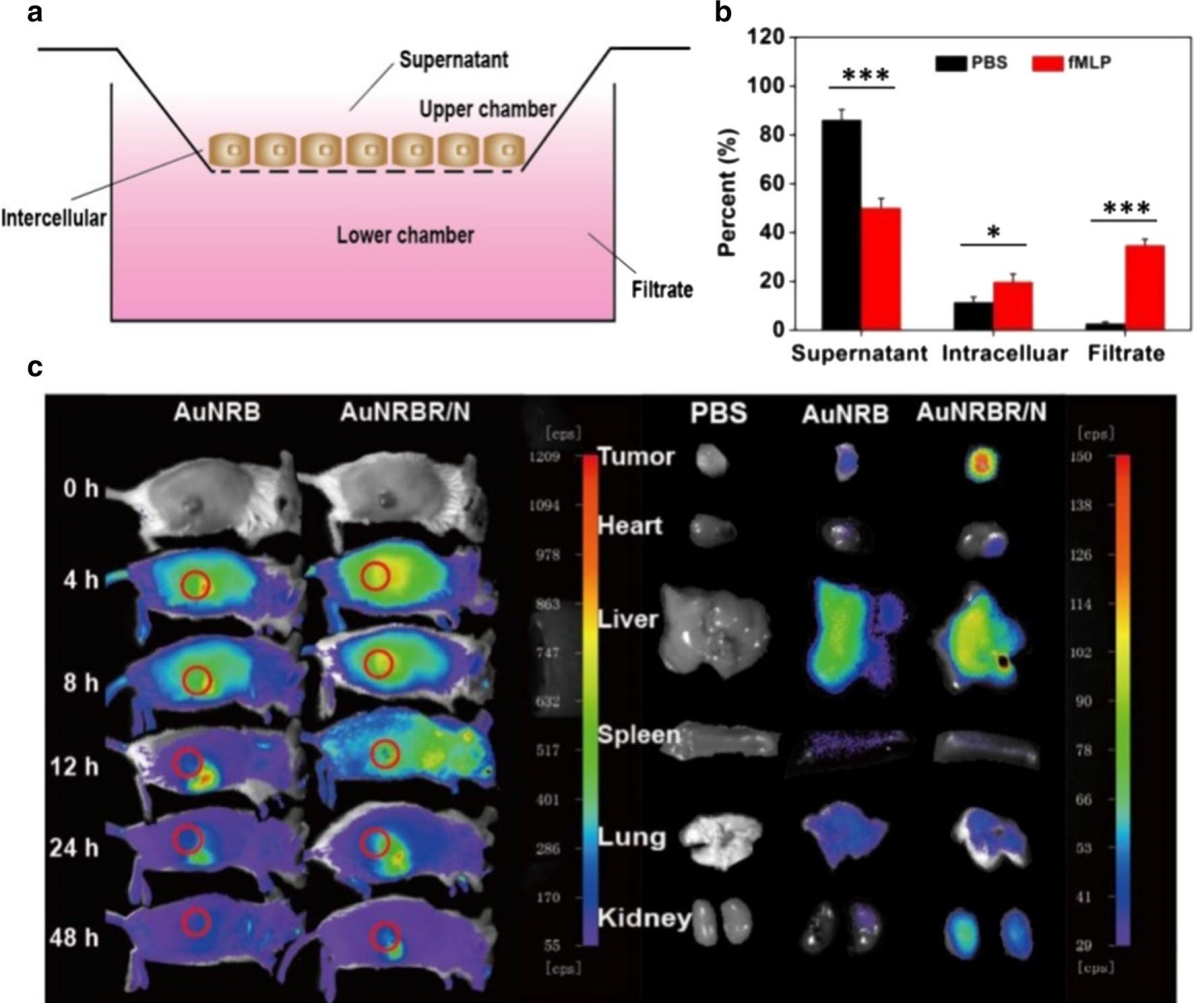
Endothelial permeability abilities of AuNRBR/N. **a** Schematic diagram for measuring penetration efficiency of AuNRBR/N across an endothelial monolayer by using a transwell system. AuNRBR quantitatively determined in the supernatant (upper chamber), cell monolayer and filtrate (lower chamber). **b** The quantity of AuNRBR distributed in each compartment in the absence or presence of fMLP for 4 h. **c** In vivo tumor bearing mice fluorescence imaging at post-injection of 4, 8, 12, 24 and 48 h (left) and major organs and tumors taken out for further imaging at post-injection of 12 h (right). The error bars were presented as mean ± SD (n = 3). *p < 0.05, ***p < 0.001

The microvascular endothelial cell penetrating ability of AuNRBR/N was studied by using a transwell technology (Fig. 5a) and by using the HUVEC as the cell monolayer. As shown in Fig. 5b, AuNRBR/N exhibited lower permeability (2%) through the monolayer in the absence of fMLP, and nearly all AuNRBR/N stayed in the upper chamber (86%), thereby suggesting that AuNRBR/N failed to pass through the endothelium layer efficiently. However, the amount of AuNRBR increased up to about 38% in the presence of fMLP, along with a decreased percentage of AuNRBR in the upper chamber (50%). The results suggested that AuNRBR/N could be recruited by inflammatory factor and then migrate across blood vessels into the tumour site efficiently.

Next, to evaluate the elevated tumour targeting efficiency and endothelial permeability of AuNRBR/N and AuNRBR in vivo, AuNRBR/N and AuNRBR were intravenously injected into Lewis tumour-bearing mice and real-time fluorescence imaging technique to detect the fluorescence intensity in tumor. Compared with AuNRBR, the area of tumour-bearing mouse administered with AuNRBR/N displayed significant fluorescence signal at 12 h post-injection. As time went on, the signal began to degrease, and the signal of both groups at 12 h post-injection was stronger than that at 24 and 48 h (Fig. 5c, left). These direct images of major organs and tumours at 24 h post-injection had a similar tendency with that presented above (Fig. 5c, right). Although AuNRBR group could target tumour to some degree due to the RGD peptide, AuNRBR/N showed enhanced tumour targeting efficiency, which originated from the optimal combination and tumour-homing ability of neutrophils recruited by chemotactic factor. This result suggested that AuNRBR/N has potent tumour targeting ability.

**Fig. 6:**
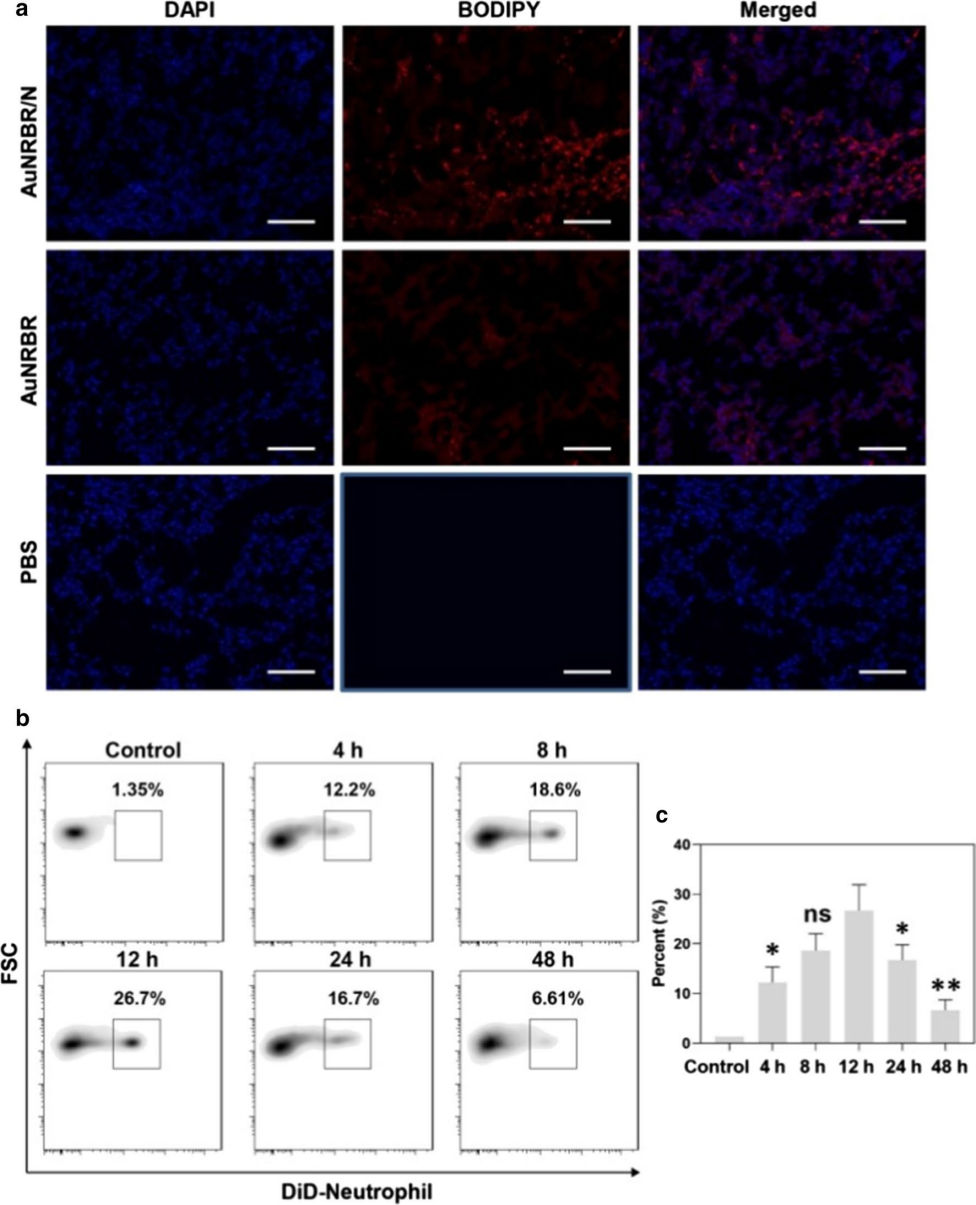
**a** CLSM images of tumor tissues in the Lewis tumor-bearing mice after injection of AuNRBR/N, AuNRBR and PBS. Blue fluorescence: nuclei, red fluorescence: BODIPY. The ratio of administered neutrophils in the total neutrophils in tumor after intravenous injection of AuNRBR/N labeled with DiD at 4, 8, 12, 24 and 48 h. The percent showed by **b** representative flow cytometry data and **c**individual histograms. Scale bar: 100 μm.*p < 0.05 and **p < 0.01 vs the 12 h group

The specific targeting of AuNRBR/N to the tumour was further investigated by CLSM. As shown by Fig. 6a, the slice of tumour-bearing mice injected with AuNRBR/N exhibited stronger fluorescence intensity than that of AuNRBR, suggesting that AuNRBR/N could be efficiently delivered to the tumour tissue due to the inflammation-mediated neutrophil migration. The weak fluorescence signal of AuNRBR group still illustrated RGD peptide-mediated active targeting.

To obtain the dynamics of AuNRBR/N in tumour after intravenous injection, the tumours were taken out and digested, followed by staining with PE/Cy7-conjugated anti-mouse Ly-6G/Ly-6C to analyse by flow cytometry. As seen from Fig. 6b, AuNRBR/N could be significantly recruited into tumours, and around 30% of neutrophils in the tumour originated from AuNRBR/N at 12 h post-injection. The ratio of exogenously injected neutrophils decreased after 12 h and descended to around 5% at 48 h post-injection (Fig. 6c). The AuNRBR/N could be effectively recruited into the tumour and eliminated gradually.

### AuNRBR/N induce potent antitumour therapy in vivo

**Fig. 7:**
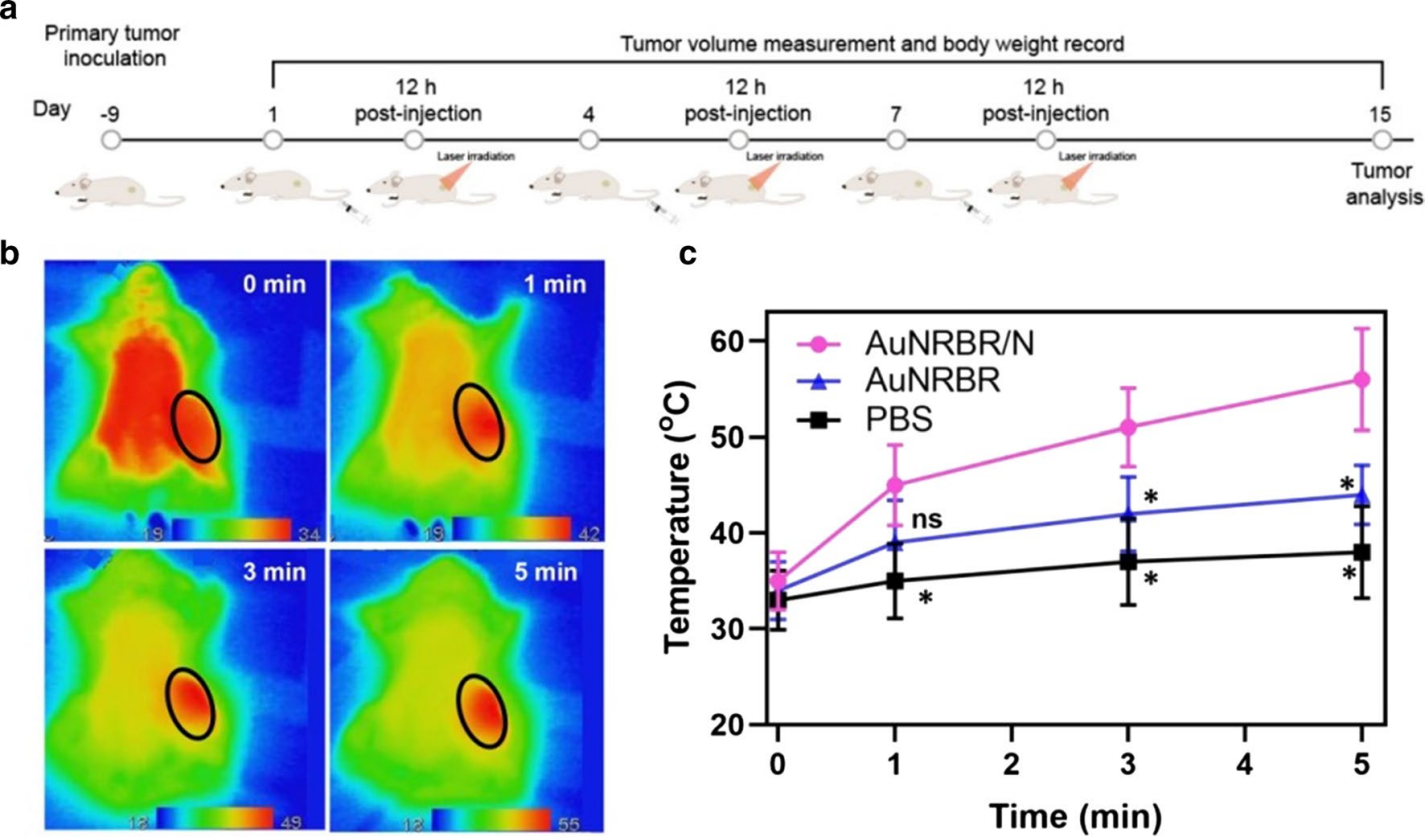
**a** Schematic diagram of the experimental design. **b** IR thermal images and **c** temperature changing curve of Lewis tumor bearing mice treated with AuNRBR/N under 808 nm laser irradiation within 5 min (0.5 W/cm^2^). *p < 0.05 vs the AuNRBR/N group

AuNR based PTT drug delivery system can induce apoptosis or necrosis of the cancer cells and suppress tumor growth by generating a localized hyperthermia effect. However, the lack of specificity of the AuNR would lead to inevitable damage to surrounding healthy tissues. The antitumour therapy experiment was designed as a schematic diagram shown in Fig. 7a. Firstly, the temperature of the Lewis tumour was detected with laser irradiation after administration of AuNRBR or AuNRBR/N. As shown in Fig. 7b, the temperature of the Lewis tumour increased up to 55 °C within 5 min when exposed to the 808 nm laser irradiation (0.5 W/cm^2^), thereby exhibiting a stronger photothermal therapeutic efficiency of AuNRBR/N to the tumour. Interestingly, AuNRBR/N showed a more significantly enhanced photothermal effect than AuNRBR, which induced the temperature increase to around 42 °C within 5 min (Fig. 7c). The elevated temperature confirmed the PTT efficiency of AuNRBR/N.

**Fig. 8:**
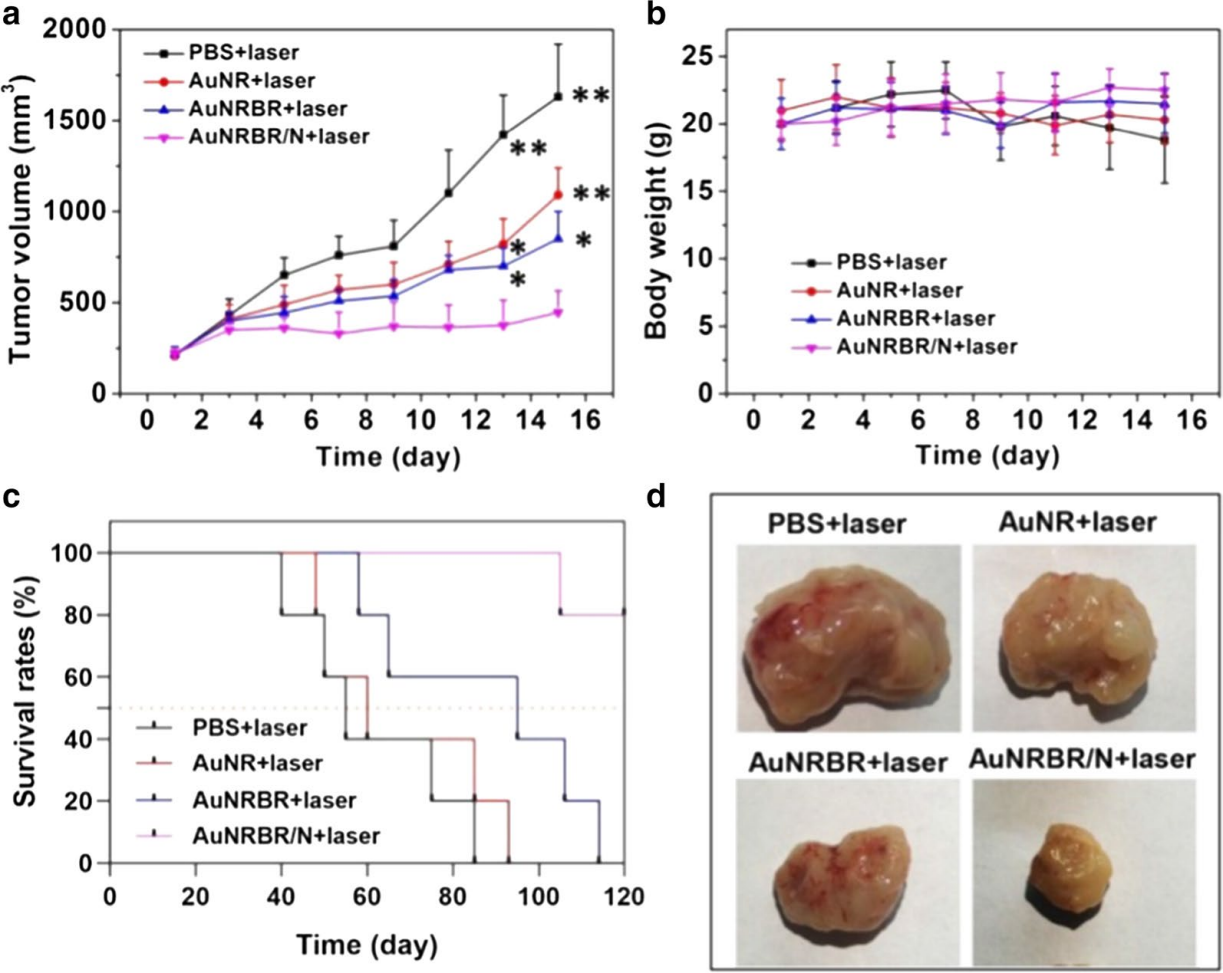
**a** Tumor growth curve and **b** body weight change of mice with different treatments during 15 days. **c** Survival curves of Lewis tumor-bearing mice after various treatments. AuNRBR/N injected mice showed improved survival over 120 days after treatments. **d** The images of tumor in mice on day 15 after various treatments. Scale bar: 100 μm. The error bars were presented as mean ± SD (n = 5).*p < 0.05 and **p < 0.01 vs the AuNRBR/N + laser group

**Fig. 9:**
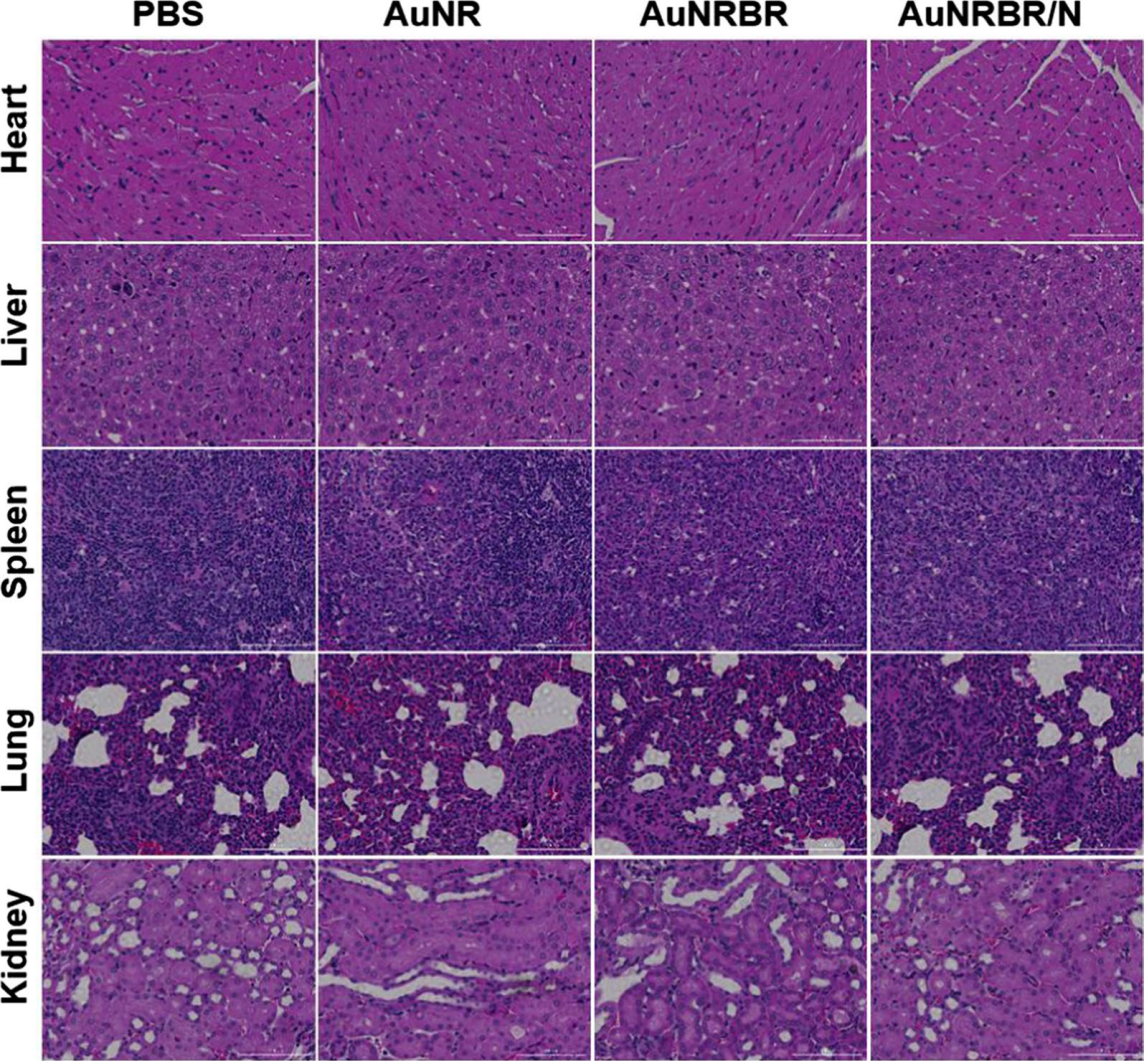
The HE staining of main organ sections at day 15 after administration of different treatments. Scale bar: 100 µm

Then, the sizes of tumours were continuously monitored for 15 days. As shown in Fig. 8a, b, the tumour volume of mice injected with PBS quickly increased, which indicated that laser irradiation alone had no therapeutic effect on the tumour. However, the tumour of AuNR- treated mice was inhibited modestly compared with PTT alone due to limited EPR effect. Moreover, the tumour growth was obviously delayed when the mice were administrated with AuNRBR, thereby suggesting the active targeting ability of AuNRBR. Apparently, complete suppression of the growth of tumour treated with AuNR or AuNRBR was difficult, thereby indicating the limited targetability, which contributed to the undesired therapeutic effect. However, the tumour growth of mice was significantly inhibited when injected with AuNRBR/N. The enhanced therapeutic effect for AuNRBR/N was ascribed to the remarkable targeting efficiency of neutrophil, thereby leading to a higher amount of AuNRBR/N recruited into the tumour. AuNRBR/N would further release AuNRBR for penetration into deep tumour tissue, thereby leading to a robust therapeutic effect. The body weight was also stable during the therapy, which suggested the biosafety of AuNRBR/N (Fig. 8c). As seen in Fig. 8d, the median survival increased from 55 days for the control group to 60 days for the AuNR group. A more significant increase in the median survival (95 days) was observed in the AuNRBR group. For the AuNRBR/N-treated group, the median survival reached over 120 days. The administration of AuNRBR/N led to a significant increase in median survival of Lewis tumour-bearing mice. Thus, AuNRBR/N can significantly inhibit the growth of the tumour due to the neutrophil-based multistage delivery system and the improved active targeting of AuNRBR to the tumour. To study the potential toxicity, major organs (heart, liver, spleen, lung and kidney) were stained with hematoxylin and eosin (H&E). As shown in Fig. 9, no significant microscopic lesions were revealed in all three groups during 15 days compared with the control group, indicating that AuNRBR/N was biocompatible and safe.

## Conclusions

We developed a novel cell-based multistage nanoparticle delivery system for PTT of cancer. The AuNR was successfully synthesised and modified with BSA. AuNRB modified with RGD can increase the targeting efficiency and evoke the intensive antitumour capability in vitro, which may increase penetration in tumour site after neutrophil delivering AuNRBR. Additionally, AuNRBR/N could migrate across the epithelial cells efficiently, had better toxicity against Lewis cells in vitro and enhanced tumour targeting efficiency in vivo. More importantly, AuNRBR/N exhibited superior therapeutic effect on Lewis tumour due to the optimal tumour targeting of neutrophils and multistage delivery of AuNRBR for deep tumour diffusion, which also improved the survival rate of mice (over 120 days). We hope this novel cell-based multistage AuNR delivery system can provide a new strategy for cancer PTT.

## Abbreviations

PTT: Photothermal therapy
AuNR: Au nanorod
NIR: Near-infrared
MPS: Mononuclear phagocytic system
FBS: Foetal bovine serum
DAPI: 4,6-diamidino-2-phenylindole
BSA: Bovine serum albumin
CTAB: Hexadecyl trimethyl ammonium bromide
DMSO: Dimethyl sulfoxide
FITC: Fluorescein5(6)-isothiocyanate
CLSM: Confocal laser scanning microscopy
HUVECs: Human umbilical vein endothelial cells
