# Correction: Neutrophils mediated multistage nanoparticle delivery for prompting tumor photothermal therapy

**DOI:** 10.1186/s12951-026-04717-3

**Published:** 2026-06-30

**Authors:** Bo Ye, Bao Zhao, Kun Wang, Yilong Guo, Qinguo Lu, Longpo Zheng, Ang Li, Jianou Qiao

**Affiliations:** 1 Department of Thoracic Surgery, Shanghai Chest Hospital, Shanghai Jiao Tong University, Shanghai, China; 2 Department of Otorhinolaryngology Head and Neck Surgery, First Affiliated Hospital of Bengbu Medical College, Bengbu, China; 3 Cancer Center, Shanghai East Hospital, Tongji University, Shanghai, China; 4 Department of Thoracic Surgery, Pizhou people’s hospital, Xuzhou Medical University, Pizhou, China; 5 Shanghai Tenth People’s Hospital, School of Medicine, Tongji University, Shanghai, 200072 People’s Republic of China; 6 School of Life Science and Technology, Tongji University, 1239 Siping Road, Shanghai, 200092 People’s Republic of China; 7 Department of Respiratory Medicine, Shanghai Ninth Peoples Hospital, Shanghai Jiao Tong University School of Medicine, 639 Zhizaoju Rd, Shanghai, 200011 China

**Correction: J Nanobiotechnol (2020) 18:138**

10.1186/s12951-020-00682-7

In Fig. 9, the HE staining image of the lung tissue has appeared incorrectly and it has now been corrected in the original publication. For completeness and transparency, the old incorrect and correct versions are displayed below.

Incorrect Fig. 9.


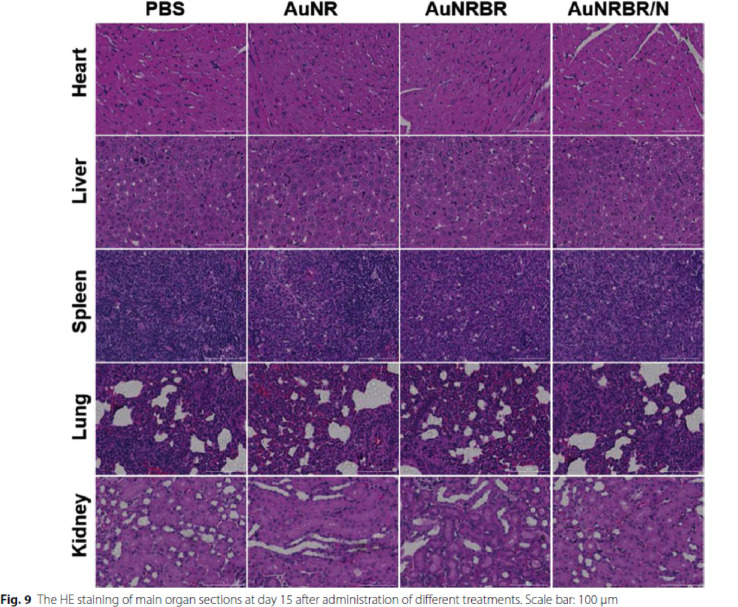


Correct Fig. 9. 

**Fig. 9:**
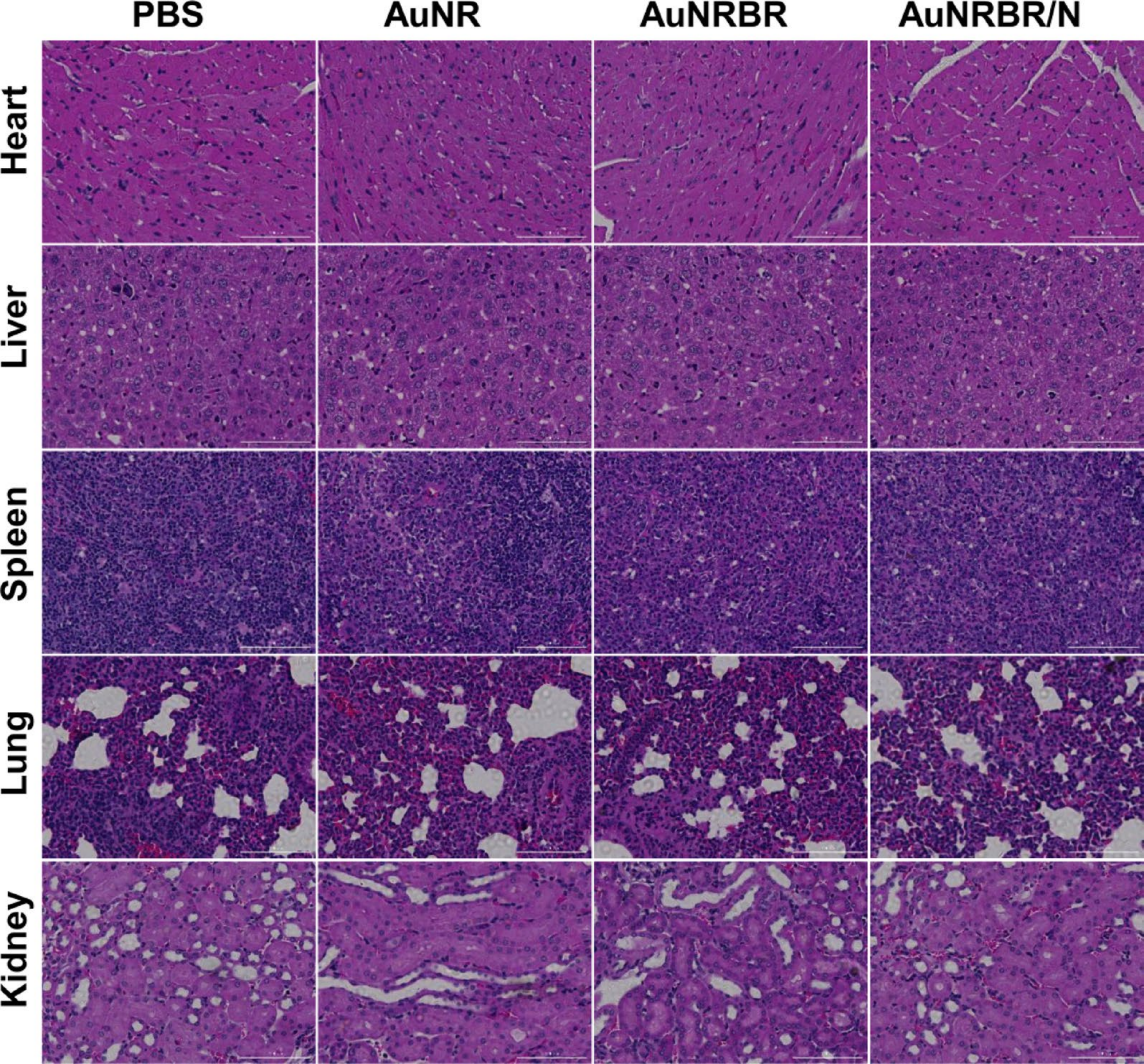
The HE staining of main organ sections at day 15 after administration of different treatments. Scale bar: 100 μm

The original article has been corrected.

